# Robotic Impedance Learning for Robot-Assisted Physical Training

**DOI:** 10.3389/frobt.2019.00078

**Published:** 2019-08-27

**Authors:** Yanan Li, Xiaodong Zhou, Junpei Zhong, Xuefang Li

**Affiliations:** ^1^Department of Engineering and Design, University of Sussex, Brighton, United Kingdom; ^2^Beijing Institute of Control Engineering, Beijing, China; ^3^School of Science and Technology, Nottingham Trent University, Nottingham, United Kingdom; ^4^Department of Electrical Engineering, Imperial College of Science, Technology and Medicine, London, United Kingdom

**Keywords:** impedance learning, impedance control, iterative learning control, physical human-robot interaction, robotic control

## Abstract

Impedance control has been widely used in robotic applications where a robot has physical interaction with its environment. However, how the impedance parameters are adapted according to the context of a task is still an open problem. In this paper, we focus on a physical training scenario, where the robot needs to adjust its impedance parameters according to the human user's performance so as to promote their learning. This is a challenging problem as humans' dynamic behaviors are difficult to model and subject to uncertainties. Considering that physical training usually involves a repetitive process, we develop impedance learning in physical training by using iterative learning control (ILC). Since the condition of the same iteration length in traditional ILC cannot be met due to human variance, we adopt a novel ILC to deal with varying iteration lengthes. By theoretical analysis and simulations, we show that the proposed method can effectively learn the robot's impedance in the application of robot-assisted physical training.

## 1. Introduction

With recent development of mobile intelligence, it has seen a clear trend that robots will come into interact with humans in industries, health care, medical applications, etc. (Ajoudani et al., 2018). Among various types of human-robot interaction, physical human-robot interaction (pHRI) has been actively studied in the past decades by researchers in robotics and human motor control (Haddadin and Croft, 2016).

It is widely acknowledged that impedance control is one of the most effective and robust approaches for pHRI, which differentiates from position control and force control by developing a relationship between the position and interaction force (Hogan, 1985). Such a relationship is usually represented by a desired or target impedance model that enables a robot to behave like a mass-damper-spring system (Albu-Schäffer et al., 2007). Therefore, a robot's dynamics under impedance control are effectively affected by impedance parameters that in general include mass/intertia, damping and stiffness components. While impedance control can be realized passively (through hardware design) (Vanderborght et al., 2013; Wu and Howard, 2018) and actively (through software) (Lippiello et al., 2018), the latter provides feasibility to adapt impedance parameters which is essential in many situations. Indeed, constant impedance parameters cannot fulfill requirements in a task where the robot's environment dynamically changes. In Ganesh et al. (2010), a robot manipulator increases its impedance when there is an external disturbance and decreases it when the disturbance vanishes to save its control effort. In Kim et al. (2010), a robot gripper that is catching a flying object needs to be compliant in the direction in which the object is moving but stiff in another direction to hold its position. In Dimeas et al. (2019), a variable stiffness controller is designed for pHRI where the robot's stiffness increases when a new path is close to the previous ones and otherwise decreases to allow the human operator to adjust. Therefore, predefining constant impedance parameters is impractical, if not impossible, to achieve the task objectives in impedance control.

Researchers have attempted impedance adaptation and impedance learning using various techniques. One group of works measure humans' impedance and use this knowledge to adapt the robot's impedance. In Rahman et al. (2002), human's impedance is estimated so that the robot can adapt its own impedance to improve their collaborative manipulation. In Erden and Billard (2015), human's hand impedance is measured for the robot to assist the human in manual welding. These approaches are model-based as they require estimation of the human's impedance parameters, so their quality relies on accurate modeling of the human. Another group of works on impedance learning are based on reinforcement learning (RL), which appreciate a fact that in many situations the robot's environment (including humans) is difficult to model. In Kim et al. (2010), natural actor-critic algorithm is used to learn impedance parameters to maximize a given reward function. In Buchli et al. (2011), another RL algorithm named policy improvement with path integrals (PI^2^) is used to develop variable impedance control. These works can be very useful in applications where a training phase is allowed or sufficient training data are available. However, in some pHRI applications, these conditions may be invalid as the human-in-the-loop means that the interaction can be gradually improved but needs to be constantly safe and efficient. The third group of works use an idea of transferring humans' impedance skills to the robot. In particular, researchers have developed various HRI interfaces for a robot to learn the humans' impedance (Ajoudani, 2015; Yang et al., 2018). These works present interesting results for specific applications, but how they can be generalized to other applications is not clear.

While not aiming at providing a general solution to impedance learning for pHRI, in this paper we focus on a specific scenario of robot-assisted physical training. Our idea is to adopt learning control in the field of control engineering to pHRI, so that the system stability and performance can be continuously evaluated. A potential difficulty is that human's behavior is hard to be modeled, so a model-free method is preferred. Another point of consideration is that physical training usually involves a repetitive process, which allows us to improve the robot's control strategy gradually. Based on these discussions, iterative learning control (ILC) has been chosen as a proper tool to fix the problem under study. In particular, ILC has been adequately studied in control engineering (Arimoto, 1990; Xu and Yan, 2006; Huang et al., 2013; Chu et al., 2016), which does not require the model knowledge but uses the historical information (in the case of pHRI, data collected from previous interactions). The use of ILC for impedance learning has proven to be successful in existing works (Tsuji and Morasso, 1996; Yang et al., 2011; Li and Ge, 2014; Li et al., 2018; Fernando et al., 2019; Ting and Aiguo, 2019), where a general robot's environment is studied. However, these works did not consider a challenge that is specially present in pHRI: due to a human individual's variance and uncertainty, one cannot guarantee that the interaction can be repeated with a fixed period. In physical training, a human partner may interact with a robot for a time duration that changes in a different iteration, even if the human partner tries their best to follow pre-set guidance. In this paper, we will address this issue by employing a recent work on ILC for iterations with time-varying lengths (Li et al., 2015; Shen and Xu, 2019). We will show that impedance learning for physical training is achieved despite that the human partner cannot repeat a motion with a fixed period. We will also elaborate how the proposed method provides assistance-as-needed, which is an important property that has proved to be useful for human patients' learning (Emken et al., 2007). We will rigorously prove the convergence of the proposed learning method, and simulate different human behaviors to demonstrate its features. The contributions of our work compared to the existing ones are 2-fold: the first is the formulation of a robotic physical training problem as an ILC problem with varying lengths; second, the design of the learning law and stability analysis are different from Shen and Xu (2019). In Shen and Xu (2019), a general system is considered and the system function is locally Lipschitz continuous with respect to the state, while the robot's dynamics are considered with specific properties in this paper.

The remainder of this paper is organized as follows. In section 2, the description of a pHRI system is given and the problem to be studied is formulated. In section 3, an impedance learning method is derived and its convergence is proved. In section 4, various human behaviors in physical training are simulated to demonstrate the features of the proposed method. In section 5, conclusions of this work are drawn.

## 2. Problem Formulation

**Figure 1:**
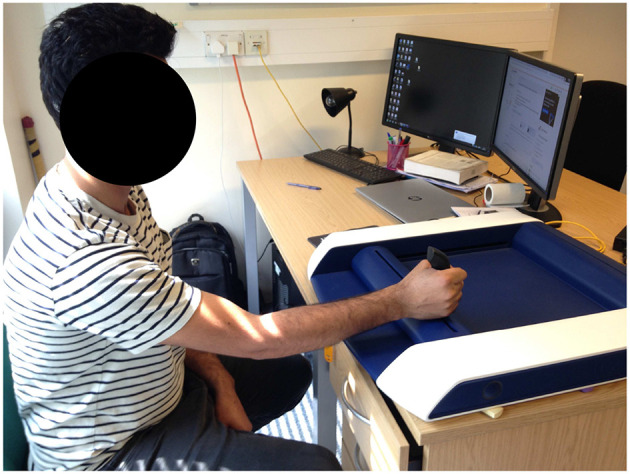
Robot-assisted upper-limb physical training. A human hand holds a handle on a planar robotic platform, while the robot is moved by its embedded motors and also by the human hand to reach a target position or track a desired trajectory.

In this paper, we consider a case of robot-assisted physical training for upper-limbs, where a human arm is attached to a robot platform (see Figure 1). The robot is driven by its embedded motors and also moved by the human arm to reach a target position or to follow a desired trajectory. In this section, we will first establish the system model, including robot's controller and human's control input. Then, we will elaborate the learning control problem that one needs to address for the robot to provide desired assistance-as-needed to the human, subject to human's unknown control input.

### 2.1. System Description

The dynamics of a robot in the joint space can be described as

(1) $$M_{j}(q)\ddot{q}+C_{j}(q,\dot{q})\dot{q}+G_{j}(q)=\tau +J^{T}(q)u_{h}$$

where *q* is the coordinate in the joint space, *M*_*j*_(*q*) is the inertia matrix, $C_{j}\left(q,\overset{∙}{q}\right)\overset{∙}{q}$ is the Coriolis and centrifugal force, *G*_*j*_(*q*) is the gravitational torque, τ is the joint torque supplied by the actuators, *u*_*h*_ is the force applied by the human and *J*(*q*) is the Jacobian matrix which is assumed to be non-singular in a finite workspace. ^*T*^ stands for the transpose of a matrix or a vector. Through kinematic transformation, the dynamics of the robot in the task space can be obtained as

(2) $$M(q)\ddot{x}+C(q,\dot{q})\dot{x}+G(q)=u+u_{h}$$

where *x* is the position/orientation of the robot's end-effector in the task space, and *M*(*q*), $C\left(q,\overset{∙}{q}\right)$, *G*(*q*), and *u* are respectively obtained as

(3) $$\begin{array}{l} M(q)=J^{-T}(q)M_{j}(q)J^{-1}(q)\\ C(q,\dot{q})=J^{-T}(q)(C_{j}(q,\dot{q})-M(q)J^{-1}(q)\dot{J}(q))J^{-1}(q)\\ G(q)=J^{-T}(q)G_{j}(q),\;u=J^{-T}(q)\tau \end{array}$$

Property 1. *(Ge et al., 1998) The matrix $2C\left(q,\overset{∙}{q}\right)-Ṁ\left(q\right)$ is a skew-symmetric matrix if $C\left(q,\overset{∙}{q}\right)$ is in the Christoffel form, i.e., $\rho^{T}\left(2C\left(q,\overset{∙}{q}\right)-Ṁ\left(q\right)\right)\rho =0$, where ρ is an arbitrary matrix with a proper dimension*.

This specific property of robot's dynamics is usually used to prove the closed-loop system stability, as will be carried out in the next section.

### 2.2. Problem Statement

From the above subsection, it is clear to see that the robot's motion is determined by both the robot's and human's control inputs *u* and *u*_*h*_. Therefore, how to design the robot's control input *u* depends on the human's control input *u*_*h*_. As the control objective is to reach a target position or to track a desired trajectory, the robot could take *u*_*h*_ as a disturbance and design a high-gain controller. However, this scheme is not desired for a physical training robot as it does not actively encourage the human to learn to complete the task by themselves. Instead, the robot needs to understand how much the human can achieve in tracking the reference trajectory and provide assistance only as much as needed. As physical training usually involves a repetitive process, iterative learning control or repetitive control may be used to achieve this objective (Cheah and Wang, 1998; Yang et al., 2011; Li et al., 2018).

For this purpose, the human's control input can be constructed based on certain periodic parameters, as follows

(4) $$u_{h}=-K_{h1}(x-x_{d})-K_{h2}\dot{x}$$

where *K*_*h*1_ and *K*_*h*2_ are the human's stiffness and damping parameters, respectively and *x*_*d*_ is a desired trajectory that is defined for a task. The above equation shows that the human can complete the tracking task to some extent, and their performance is determined by their control parameters *K*_*h*1_ and *K*_*h*2_ which are unknown to the robot. For the robot to learn these parameters, it is assumed that they are iteration-invariant, i.e.,

(5) $$K_{h1}^{i}(t)=K_{h1}^{i-1}(t),\;K_{h2}^{i}(t)=K_{h2}^{i-1}(t)$$

where *i* is the iteration number and *t* ∈ [0, *T*] with *T* as a fixed time duration.

In the rest of the paper, the subscript ^*i*^ is omitted where no confusion is caused. Different from traditional iterative learning control where each iteration lasts for a fixed time duration *T*, here we assume that each iteration can have a different length *T*^*i*^. This is a necessary assumption in robot-assisted physical training, where it is difficult to require the human to repeat a motion within exactly the same time duration: in one iteration, the human may complete the motion in a shorter time duration, while in another iteration, the human may need more time to complete the motion.

This uncertainty from the human raises an issue to the robot's controller design. If *T*^*i*^ < *T*, there are no data between *T*^*i*^ and *T* that can be used for learning in the next iteration; if *T*^*i*^ > *T*, how to use the data beyond *T* for learning needs to be studied. In this paper, we aim to address this problem to enable effective learning for robot-assisted physical training. Without loss of generality, we assume that *T*^*i*^ ≤ *T* for all iterations, where *T* is known. In practice, *T* can be set large enough to cover all possible iterations.

## 3. Approach

In this section, we develop the robot's controller that assists the human partner in tracking a desired trajectory, while it evaluates the human partner's performance by iterative learning.

### 3.1. Robot's Controller

The robot's control input *u* is designed as below

(6) $$u\text{ = }u_{1}+u_{2}$$

where *u*_1_ is used to compensate for the dynamics of robot and *u*_2_ is dedicated to learning the human's control input and determining the robot's impedance.

How to design *u*_1_ has been studied extensively in the literature, such as adaptive control (Slotine and Li, 1987), neural networks control (Ge et al., 1998), etc. In this paper, to align with the learning design, we develop *u*_1_ as below

(7) $$u_{1}={\hat{\theta }}^{T}Y({\ddot{x}}_{e},{\dot{x}}_{e},\dot{q},q)-L\varepsilon$$

where $Y\left({ẍ}_{e},{ẋ}_{e},\overset{∙}{q},q\right)$ is a known regressor and $\hat{\theta }$ is an estimate of θ that represents the physical parameters in the system's dynamic model, as below

(8) $$M(q)\ddot{x}+C(q,\dot{q})\dot{x}+G(q)=\theta^{T}Y(\ddot{x},\dot{x},\dot{q},q)$$

ẋ_*e*_ is an auxiliary variable that is defined as

(9) $${\dot{x}}_{e}={\dot{x}}_{d}-\alpha e$$

where *e* = *x* − *x*_*d*_ is the tracking error and *α* is a positive scalar. *L* is a positive-definite matrix and *ε* is an auxiliary error defined as

(10) $$\varepsilon =\dot{e}+\alpha e.$$

The learning part in the robot's controller *u*_2_ is designed as

(11) $$u_{2}={\hat{K}}_{h1}e+{\hat{K}}_{h2}\dot{x}$$

where ${\hat{K}}_{h1}$ and ${\hat{K}}_{h2}$ are estimates of *K*_*h*1_ and *K*_*h*2_ in Equation (4), respectively. They are also the robot's impedance parameters, i.e., stiffness and damping, respectively.

By substituting the robot's controller in Equation (6) [with Equations (7) and (11)] and the human's control input in Equation (4) into the robot dynamics in Equation (2), we obtain the error dynamics as below

(12) $$M\dot{\varepsilon }+C\varepsilon ={\tilde{\theta }}^{T}Y({\ddot{x}}_{e},{\dot{x}}_{e},\dot{q},q)-L\varepsilon +{\tilde{K}}_{h1}e+{\tilde{K}}_{h2}\dot{x}$$

where

(13) $$\tilde{\theta }=\hat{\theta }-\theta ,\;{\tilde{K}}_{h1}={\hat{K}}_{h1}-K_{h1},\;{\tilde{K}}_{h2}={\hat{K}}_{h2}-K_{h2}$$

The above error dynamics indicate that the error *ε* is due to the estimation errors $\tilde{\theta }$, ${\tilde{K}}_{h1}$ and ${\tilde{K}}_{h2}$. Therefore, learning laws of $\hat{\theta }$, ${\hat{K}}_{h1}$ and ${\hat{K}}_{h2}$ need to be developed to eliminate these estimation errors.

### 3.2. Learning Law

In this subsection, we discuss how to design the learning laws to obtain the estimated parameters $\hat{\theta }$, ${\hat{K}}_{h1}$, and ${\hat{K}}_{h2}$. For this purpose, some preliminaries and assumptions for ILC are needed.

Assumption 1. *At the beginning of each iteration, the actual position is reset to the initial desired position, i.e., *x*(0) = *x*_*d*_(0)*.

The identical initial condition is applied to ensure perfect tracking in ILC. Due to the fact that it may be difficult to reset the initial state to a same value in practice, some efforts have been made to relax this condition in ILC area, such as Chen et al. (1999). In the context of this work, the identical initial condition is assumed for simplicity purpose but it would not be difficult to incorporate the techniques in Chen et al. (1999) with the proposed controller if the resetting condition cannot be guaranteed.

Then, according to ILC with randomly varying iteration lengths in Shen and Xu (2019), a virtual position error is defined as below

(14) $$\bar{\varepsilon }(t)=\{\begin{array}{ll} \varepsilon (t), & t\leq T^{i};\\ \varepsilon (T^{i}), & T^{i}<t\leq T. \end{array}$$

The above definition indicates that the missing error beyond *T*^*i*^ is supplemented by the error at time *t* = *T*^*i*^.

Assumption 2. *T*^*i*^ *is a random variable that has a range of [*T*_0_, *T*] where *T*_0_ > 0. When the iteration number *i* → ∞, there are infinite iterations with* *T*^*i*^ = *T*.

The above assumption implies that although *T*^*i*^ is subject to a certain probabilistic distribution, it will reach the maximum length *T* for many times when the iteration number is large enough.

In Shen and Xu (2019), a general system is considered where the system function is locally Lipschitz continuous with respect to the state. In this paper, the robot's dynamics are considered with specific properties so the design of the learning laws is different. In particular, a virtual mass/intertia matrix is defined as

(15) $$\bar{M}(q)=\{\begin{array}{ll} M(q(t)), & t\leq T^{i};\\ M(q(T^{i})), & T^{i}<t\leq T. \end{array}$$

In order to show the convergence of ILC, let us consider a composite energy function

(16) $$\begin{array}{l} \;E(t)=E_{1}(t)+E_{2}(t),\\ E_{1}(t)=\frac{1}{2}\bar{\varepsilon }{\left(t\right)}^{T}\bar{M}\left(q\right)\bar{\varepsilon }\left(t\right),\\ E_{2}(t)=\text{tr}[\frac{1}{2\beta_{\theta }}\int_{0}^{t}{\tilde{\theta }}^{T}(\tau )\tilde{\theta }(\tau )d\tau \\ \;+\frac{1}{2\beta_{1}}\int_{0}^{t}{\tilde{K}}_{h1}^{T}(\tau ){\tilde{K}}_{h1}(\tau )d\tau +\frac{1}{2\beta_{2}}\int_{0}^{t}{\tilde{K}}_{h2}^{T}(\tau ){\tilde{K}}_{h2}(\tau )d\tau ] \end{array}$$

where tr(·) is the trace of a matrix, and *β*_θ_, *β*_1_, and *β*_2_ are learning rates that are set as positive scalars. The learning laws are then designed to minimize the above composite energy function iteratively, as below

(17) $$\begin{array}{l} \;{\hat{\theta }}^{i}(t)={\hat{\theta }}^{i-1}(t)-\beta_{\theta }\varepsilon (t)Y^{T}({\ddot{x}}_{e},{\dot{x}}_{e},\dot{q},q)\\ {\hat{K}}_{h1}^{i}(t)={\hat{K}}_{h1}^{i-1}(t)-\beta_{1}\varepsilon (t)e^{T}(t)\\ {\hat{K}}_{h2}^{i}(t)={\hat{K}}_{h2}^{i-1}(t)-\beta_{2}\varepsilon {\dot{x}}^{T}(t),\;t\leq T^{i} \end{array}$$

where ${\hat{\theta }}^{0}\left(t\right)=0$, ${\hat{K}}_{h1}^{0}\left(t\right)=0$ and ${\hat{K}}_{h2}^{0}\left(t\right)=0$, *t* ∈ [0, *T*]. Note that the above learning laws are driven by the error ε, which indicates that the learning will terminate if *ε* = 0. This is a property relevant to assistance-as-needed: if the human can complete the task, i.e., *ε* = 0, then the robot will not update its parameters to provide extra assistance.

Since there are no data for learning for *T*^*i*^ < *t* ≤ *T*, the learning laws become

(18) $$\begin{array}{l} \;{\hat{\theta }}^{i}(t)={\hat{\theta }}^{i-1}(t)\\ {\hat{K}}_{h1}^{i}(t)={\hat{K}}_{h1}^{i-1}(t)\\ {\hat{K}}_{h2}^{i}(t)={\hat{K}}_{h2}^{i-1}(t),\;T^{i}<t\leq T \end{array}$$

The above learning laws indicate that the parameters will not be updated if data are missing beyond *T*^*i*^ in the *i*−th iteration. This is a novel mechanism that deals with varying iteration lengths.

### 3.3. Convergence of Learning

In this section, we show that the proposed robot's controller and learning laws guarantee assistance-as-needed to the human, when the human's controller is unknown but periodic in its parameters with varying iteration lengths. The main results of the learning convergence are stated in the following theorem.

Theorem 1. *Consider the system dynamics in Equation (2) and the human's controller in Equation (4), with Assumptions 1 and 2. The robot's controller in Equation (6) [including Equations (7) and (11)] with the learning laws in Equations (17) and (18) guarantees that the error *ε* converges to zero iteratively when the iteration number *i* goes to infinity*.

*Proof*: Since the definitions of $\bar{\varepsilon }\left(t\right)$ and the learning laws are different when *t* ≤ *T*^*i*^ and *T*^*i*^ < *t* ≤ *T*, we study the change of the composite energy function *E* in two cases.

Case 1: *t* ≤ *T*^*i*^

In this case, we have $\bar{\varepsilon }\left(t\right)=\varepsilon \left(t\right)$ and $\bar{M}\left(q\right)=M\left(q\right)$ according to their definitions. Thus, the time derivative of *E*_1_(*t*) is

(19) $$\begin{array}{l} {\dot{E}}_{1}(t)=\varepsilon^{T}M\dot{\varepsilon }+\frac{1}{2}\varepsilon^{T}\dot{M}\varepsilon \\ \;=\varepsilon^{T}M\dot{\varepsilon }+\varepsilon^{T}C\varepsilon \end{array}$$

where the second equation comes from Property 1.

By substituting the error dynamics in Equation (12) into Equation (19), we obtain

(20) $${\dot{E}}_{1}(t)=\varepsilon^{T}({\tilde{\theta }}^{T}Y+{\tilde{K}}_{h1}^{T}e+{\tilde{K}}_{h2}^{T}\dot{x}-L\varepsilon )$$

where the arguments of *Y* are omitted. By integrating Ė_1_ from 0 to *t*, we obtain

(21) $$\begin{array}{l} \;\Delta E_{1}=E_{1}^{i}(t)-E_{1}^{i-1}(t)\leq E_{1}^{i}(t)\\ =\int_{0}^{t}\left[-\varepsilon^{T}L\varepsilon +\text{tr}({\tilde{\theta }}^{T}\varepsilon Y^{T})+\text{tr}({\tilde{K}}_{h1}^{T}\varepsilon e^{T})+\text{ tr}({\tilde{K}}_{h2}^{T}\varepsilon {\dot{x}}^{T})\right]\;d\tau \end{array}$$

where Δ(·) = (·)^*i*^(*t*) − (·)^*i* − 1^(*t*) is the difference between two consecutive periods with *T* and we have $E_{1}^{i}\left(0\right)=0$ according to Assumption 1.

Then, we consider the difference between *E*_2_(*t*) of two consecutive periods as below

(22) $$\begin{array}{l} \;\Delta E_{2}=E_{2}^{i}(t)-E_{2}^{i-1}(t)\\ =\int_{0}^{t}\{\text{tr}[\frac{1}{2\beta_{\theta }}{\tilde{\theta }}^{i,T}(\tau ){\tilde{\theta }}^{i}(\tau )-{\tilde{\theta }}^{i-1,T}(\tau ){\tilde{\theta }}^{i-1}(\tau )\\ \;+\frac{1}{2\beta_{1}}({\tilde{K}}_{h1}^{i,T}(\tau ){\tilde{K}}_{h1}^{i}(\tau )-{\tilde{K}}_{h1}^{i-1,T}(\tau ){\tilde{K}}_{h1}^{i-1}(\tau ))\\ \;+\frac{1}{2\beta_{2}}({\tilde{K}}_{h2}^{i,T}(\tau ){\tilde{K}}_{h2}^{i}(\tau )-{\tilde{K}}_{h2}^{i-1,T}(\tau ){\tilde{K}}_{h2}^{i-1}(\tau ))]\}d\tau \end{array}$$

By expanding the first component in the above equation, we have

(23) $$\begin{array}{l} \;\frac{1}{2\beta_{\theta }}\text{tr}[{\tilde{\theta }}^{i,T}(\tau ){\tilde{\theta }}^{i}(\tau )-{\tilde{\theta }}^{i-1,T}(\tau ){\tilde{\theta }}^{i-1}(\tau )]\\ =\frac{1}{2\beta_{\theta }}\text{tr}[{\tilde{\theta }}^{i,T}(\tau ){\tilde{\theta }}^{i}(\tau )-{\tilde{\theta }}^{i,T}(\tau ){\tilde{\theta }}^{i-1}(\tau )]\\ \;+\frac{1}{2\beta_{\theta }}\text{tr}[{\tilde{\theta }}^{i,T}(\tau ){\tilde{\theta }}^{i-1}(\tau )-{\tilde{\theta }}^{i-1,T}(\tau ){\tilde{\theta }}^{i-1}(\tau )]\\ =\frac{1}{2\beta_{\theta }}\text{tr}[{\tilde{\theta }}^{i,T}(\tau )\Delta \hat{\theta }(\tau )+{\tilde{\theta }}^{i-1,T}(\tau )\Delta \hat{\theta }(\tau )] \end{array}$$

where we have used the assumption that θ(*t*) is iteration-invariant. By substituting the learning laws in Equation (17), the above equation can be further written as

(24) $$\begin{array}{l} \;\frac{1}{2\beta_{\theta }}\text{tr}[{\tilde{\theta }}^{i,T}(\tau ){\tilde{\theta }}^{i}(\tau )-{\tilde{\theta }}^{i-1,T}(\tau ){\tilde{\theta }}^{i-1}(\tau )]\\ =\frac{1}{2\beta_{\theta }}\text{tr}[2{\tilde{\theta }}^{i,T}(\tau )-\Delta {\hat{\theta }}^{T}(\tau )]\Delta \hat{\theta }(\tau )\\ ⩽\frac{1}{\beta_{\theta }}\text{tr}[{\tilde{\theta }}^{i,T}(\tau )\Delta \hat{\theta }(\tau )]=-\text{tr}[{\tilde{\theta }}^{i,T}(\tau )\varepsilon (\tau )Y^{T}] \end{array}$$

By expanding the other components in Equation (22), we can similarly obtain

(25) $$\begin{array}{l} \frac{1}{2\beta_{1}}\text{tr}[{\tilde{K}}_{h1}^{i,T}(\tau ){\tilde{K}}_{h1}^{i}(\tau )-{\tilde{K}}_{h1}^{i-1,T}(\tau ){\tilde{K}}_{h1}^{i-1}(\tau )]\\ ⩽-\text{tr}[{\tilde{K}}_{h1}^{i,T}(\tau )\varepsilon (\tau )e^{T}(\tau )]\\ \frac{1}{2\beta_{1}}\text{tr}[{\tilde{K}}_{h2}^{i,T}(\tau ){\tilde{K}}_{h2}^{i}(\tau )-{\tilde{K}}_{h2}^{i-1,T}(\tau ){\tilde{K}}_{h2}^{i-1}(\tau )]\\ ⩽-\text{tr}[{\tilde{K}}_{h2}^{i,T}(\tau )\varepsilon (\tau ){\dot{x}}^{T}(\tau )] \end{array}$$

By substituting Inequations (24) and (25) into Equations (21) and (22), we obtain

(26) $$\Delta E=\Delta E_{1}+\Delta E_{2}\;⩽-\int_{0}^{t}\varepsilon^{T}L\varepsilon d\tau \leq 0$$

Therefore, so far we have shown that the composite energy function *E* does not increase when the iteration number increases for *t* ≤ *T*^*i*^.

Case 2: *T*^*i*^ < *t* ≤ *T*

In this case, we have $\bar{\varepsilon }\left(t\right)=\varepsilon \left(T^{i}\right)$ and $\bar{M}\left(q\right)=M\left(q\left(T^{i}\right)\right)$. Thus, according to Equation (21), we have

(27) $$\begin{array}{l} \;\Delta E_{1}=E_{1}^{i}(t)-E_{1}^{i-1}(t)\leq E_{1}^{i}(T^{i})\\ =\int_{0}^{T^{i}}[-\varepsilon^{T}L\varepsilon +\text{tr}({\tilde{\theta }}^{T}\varepsilon Y^{T})+\text{tr}({\tilde{K}}_{h1}^{T}\varepsilon e^{T})\\ \;+\text{tr}({\tilde{K}}_{h2}^{T}\varepsilon {\dot{x}}^{T})]\;d\tau \end{array}$$

On the other hand, according to the learning laws in Equation (18) and Inequations (24) and (25), we have

(28) $$\begin{array}{l} \frac{1}{2\beta_{\theta }}\text{tr}[{\tilde{\theta }}^{i,T}(t){\tilde{\theta }}^{i}(t)-{\tilde{\theta }}^{i-1,T}(t){\tilde{\theta }}^{i-1}(t)]=0\\ \frac{1}{2\beta_{1}}\text{tr}[{\tilde{K}}_{h1}^{i,T}(t){\tilde{K}}_{h1}^{i}(t)-{\tilde{K}}_{h1}^{i-1,T}(t)(t){\tilde{K}}_{h1}^{i-1}(t)]=0\\ \frac{1}{2\beta_{2}}\text{tr}[{\tilde{K}}_{h2}^{i,T}(\tau ){\tilde{K}}_{h2}^{i}(\tau )-{\tilde{K}}_{h2}^{i-1,T}(\tau )(\tau ){\tilde{K}}_{h2}^{i-1}(\tau )]=0 \end{array}$$

Therefore, the difference between *E*_2_(*t*) of two consecutive periods becomes

(29) $$\Delta E_{2}=E_{2}^{i}(t)-E_{2}^{i-1}(t)=E_{2}^{i}(T^{i})-E_{2}^{i-1}(T^{i})$$

By considering Inequation (26), we obtain

(30) $$\Delta E=\Delta E_{1}+\Delta E_{2}⩽-\int_{0}^{T^{i}}\varepsilon^{T}L\varepsilon d\tau \leq 0$$

By Inequations (26) and (30), we have shown that the composite energy function *E* does not increase when the iteration number increases for *t* ∈ [0, *T*]. Then, we will have the boundedness of *E* if *E* in the first iteration is bounded, i.e., *E*^1^ < ∞.

Let us consider the time derivative of *E*^1^ as below

(31) $${\dot{E}}^{1}={\dot{E}}_{1}+{\dot{E}}_{2}$$

According to Inequations (26), for *t* ≤ *T*^1^ we have

(32) $${\dot{E}}^{1}⩽-\varepsilon^{T}L\varepsilon \leq 0$$

Therefore, by integrating Ė^1^ from 0 to *t*, we obtain

(33) $$E^{1}-E^{1}(0)\leq 0$$

According to Assumption 1, we have *E*_1_(0) = 0 as ε(0) = 0. Since the period *T* and true values of parameters θ, *K*_*h*1_, *K*_*h*2_ are bounded and ${\hat{\theta }}^{0}\left(t\right)=0$, ${\hat{K}}_{h1}^{0}\left(t\right)=0$, ${\hat{K}}_{h2}^{0}\left(t\right)=0$, *E*_2_(0) is bounded. Therefore, *E*^1^(0) is bounded, and thus *E*^1^ is bounded.

For *T*^1^ < *t* ≤ *T*, since $E_{1}\left(t\right)=E_{1}\left(T^{1}\right)$, *E*_1_(*t*) is bounded. By integrating Ė_2_ from *T*^1^ to *t*, we obtain

(34) $$\begin{array}{l} E_{2}(t)-E_{2}(T^{1})=\text{tr}(\frac{1}{2\beta_{\theta }}\int_{T^{1}}^{t}\theta^{T}\theta d\tau +\frac{1}{2\beta_{1}}\int_{T^{1}}^{t}K_{h1}^{T}K_{h1}d\tau \\ \;+\frac{1}{2\beta_{2}}\int_{T^{1}}^{t}K_{h2}^{T}K_{h2}d\tau ) \end{array}$$

where we have considered that ${\hat{\theta }}^{1}\left(t\right)={\hat{\theta }}^{0}\left(t\right)=0$, ${\hat{K}}_{h1}^{1}\left(t\right)={\hat{K}}_{h1}^{0}\left(t\right)=0$ and ${\hat{K}}_{h2}^{1}\left(t\right)={\hat{K}}_{h2}^{0}\left(t\right)=0$ according to Equation (18). Since the time duration *T*^1^ and true values of parameters θ, *K*_*h*1_, *K*_*h*2_ are bounded, $E_{2}\left(t\right)-E_{2}\left(T^{1}\right)$ is bounded. Since $E_{2}\left(T^{1}\right)$ is bounded, *E*_2_(*t*) is bounded. Therefore, *E*^1^ is bounded.

Finally, by Inequations (26) and (30), we have

(35) $$\Delta E^{i}\;⩽\;\{\begin{array}{ll} -\int_{0}^{t}\varepsilon^{T}L\varepsilon d\tau , & t\leq T^{i};\\ -\int_{0}^{T^{i}}\varepsilon^{T}L\varepsilon d\tau , & T^{i}<t\leq T. \end{array}$$

From the above inequality, we obtain

(36) $$E^{i}-E^{1}\;⩽\;\{\begin{array}{ll} -\Sigma_{j=1}^{i-1}\int_{0}^{t}\varepsilon^{T}L\varepsilon d\tau , & t\leq T^{j};\\ -\Sigma_{j=1}^{i-1}\int_{0}^{T^{i}}\varepsilon^{T}L\varepsilon d\tau , & T^{j}<t\leq T. \end{array}$$

which leads to

(37) $$E^{1}\geq \{\begin{array}{ll} \Sigma_{j=1}^{i-1}\int_{0}^{t}\varepsilon^{T}L\varepsilon d\tau , & t\leq T^{j};\\ \Sigma_{j=1}^{i-1}\int_{0}^{T^{i}}\varepsilon^{T}L\varepsilon d\tau , & T^{j}<t\leq T. \end{array}$$

Since *E*^1^ is bounded, we can conclude that ||ε|| → 0 when the iteration number *i* → ∞. Note that this result is valid for the whole iteration of *t* ∈ [0, *T*], as there are infinite iterations with *T*^*i*^ = *T* when *i* → ∞ according to Assumption 2. This completes the proof.

## 4. Simulation

**Figure 2:**
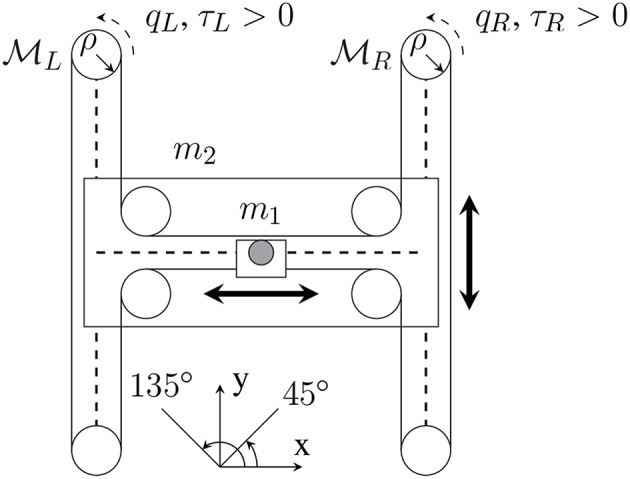
H-Man diagram. Dashed lines: linear glides. Black dot: handle, fixed to its carriage (white rectangle). White disks: pulleys. The two motors $M_{L}$ and $M_{R}$ have the same inertia *I*. *q*_*L*_, *q*_*R*_: motor rotation angles. τ_*L*_, τ_*R*_: motor torques. ρ: radius of leading pulleys. *m*_1_: mass of handle and its support. *m*_2_: mass of carriage with lateral linear guide without handle.

In this section, we simulate a robot-assisted physical training scenario, where the robot is a cable-actuated 2-DOF planar manipulandum H-Man (see Figure 2) (Campolo et al., 2014). Human and robot interact in the operational space and the common system position at the robot's handle changes due to both of their control inputs. Two tasks are considered to simulate a typical physical training process: one is to move the handle from a starting position to a target position, representing a reaching task; and the other is to track a circular reference trajectory, representing a tracking task. The target position and the reference trajectory are known to both the robot and the human.

H-Man's mass/inertial matrix is given by [*m*_1_ 0; 0 *m*_1_ + *m*_2_] where *m*_1_ = 1kg is the mass of the handle and *m*_2_=2kg is the mass of the carriage, while the motors' inertias are ignored. The mechanical friction of the H-Man is ignored and thus its *C* matrix is zero.

In each iteration, the position in the operational space is initialized as *x*(0) = [0, 0]^*T*^m. In the reaching task, the robot's reference trajectory is given as below

(38) $$x_{d}=t^{3}\left(10-15t+6t^{2}\right){\left[0,0.1\right]}^{T}$$

which indicates a smooth motion in the y direction but no motion in the x direction. In the tracking task, the robot's reference trajectory is given as below

(39) $$x_{d}={\left[0.1\sin\left(4t\right),0.1\left(1-\cos\left(4t\right)\right)\right]}^{T}$$

which indicates a circular trajectory with a radius of 0.1 m. While a complete iteration lasts for 2s, the length of each iteration is set as *T*^*i*^ = 2(1 − 0.4*rand*)s where *rand* is a function generating a random number between 0 and 1. Therefore, the time duration of each iteration is uncertain and can change from 1.2 to 2 s.

In all the simulations, the robot's parameters are set the same: *L* = 1_2_ in Equation (7) where 1_2_ is a 2 × 2 unit matrix, α = 10 in Equation (9) and β_1_ = β_2_ = 100 in Equation (17). The human's control parameters are first set as *K*_*h*1_ = −300 × 1_2_ and *K*_*h*2_ = −10 × 1_2_, simulating a human arm that deviates from the desired trajectory. They will be changed to emulate different human arms in the following sections.

### 4.1. Reaching of a Target

**Figure 3:**
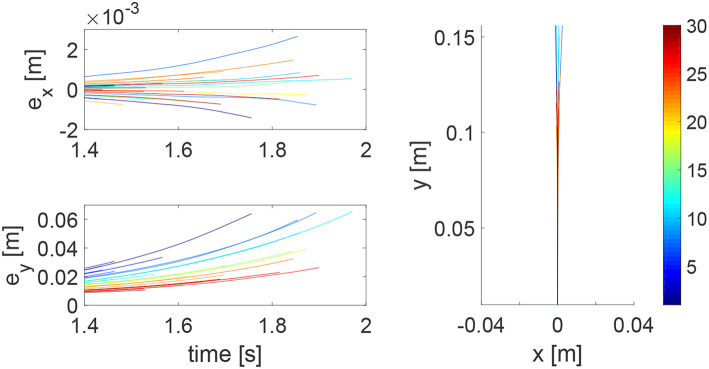
Tracking errors in the *x* direction (top left) and *y* direction (bottom left) and trajectories in the *x* − *y* plane (right column). The learning process is shown by the color bar, which changes from blue (iteration number *i* = 1) to red (*i* = 30).

**Figure 4:**
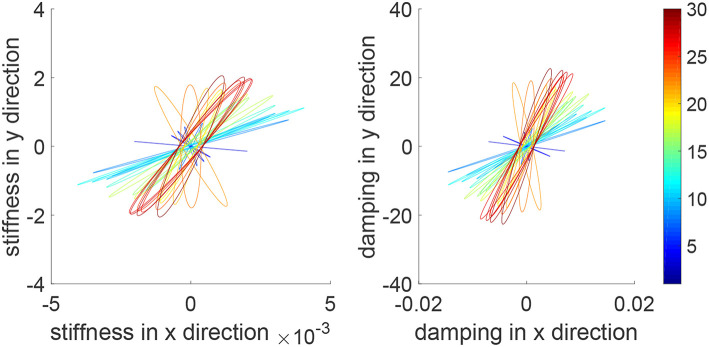
Stiffness ellipse (left) and damping ellipse (right) in all iterations. The learning process is shown by the color bar, which changes from blue (iteration number *i* = 1) to red (*i* = 30).

**Figure 5:**
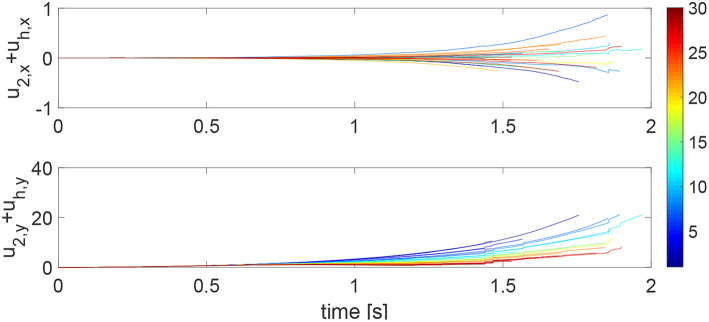
Sum of the learning part in the robot's control input and the human's control input, i.e., *u*_2_ + *u*_*h*_ in the *x* direction (top) and *y* direction (bottom). The learning process is shown by the color change from blue (iteration number *i* = 1) to red (*i* = 30).

We consider a reaching task in the first simulation, with the results in Figures 3–5. Figure 3 shows that the reaching task cannot be accomplished before the impedance learning, as the simulated human moves the robot's handle away from the desired trajectory. In particular, a large tracking error is found in the y direction. When the iteration number increases, the reaching performance is gradually improved as shown by the decreasing tracking errors in both x and y directions. Note that this is achieved despite each iteration having a different length, thus verifying the validity of the proposed learning method for repetitive processes with varying length iterations. Impedance parameters through the learning process are presented in Figure 4. To intuitively illustrate the change of impedance parameters, we plot stiffness and damping ellipses in each iteration, where the eigenvalues of stiffness and damping matrices are used as the semi-major and semi-minor axes of the respective ellipses and the eigenvectors are used to determine the angle between the major axis and x axis. It is found that the y component of these parameters is obviously larger than the x component, which is in line with the expectation as there is little motion in the x direction. Also it is important to point out that these learned impedance parameters are not necessarily equal to the human's real control parameters *K*_*h*1_ = −300 × 1_2_ and *K*_*h*2_ = −10 × 1_2_, respectively, but the robot's control input *u*_2_ in Equation (11) should ideally compensate for *u*_*h*_. As shown in Figure 5, *u*_2_ + *u*_*h*_ iteratively decreases as the iteration number increases.

**Figure 6:**
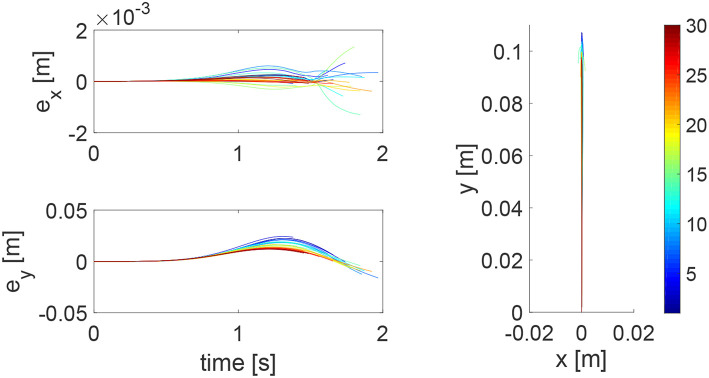
Tracking errors in the *x* direction (top left) and *y* direction (bottom left) and trajectories in the *x* − *y* plane (right column) when the human's control parameters are time-varying within an iteration. The learning process is shown by the color bar, which changes from blue (iteration number *i* = 1) to red (*i* = 30).

**Figure 7:**
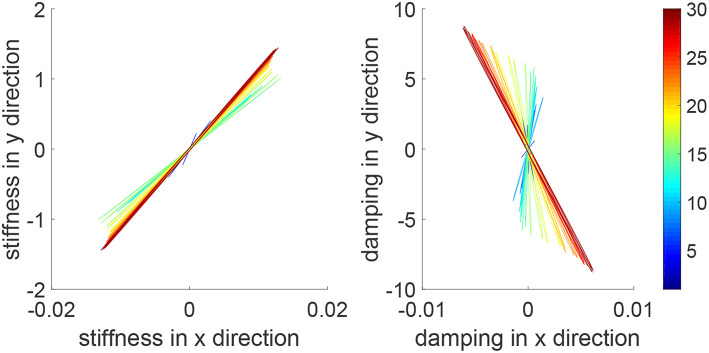
Stiffness ellipse (left) and damping ellipse (right) in all iterations when the human's control parameters are time-varying within an iteration. The learning process is shown by the color bar, which changes from blue (iteration number *i* = 1) to red (*i* = 30).

During an iteration, the human arm's control parameters may be time-varying. To simulate this situation, we set $K_{h1}=-300\sin\left(\frac{\pi }{2}t\right)\times 1_{2}$. The tracking errors and position profiles are shown in Figure 6 and impedance parameters are shown in Figure 7. It is found that these results are similar to that in Figures 3, 4, as the learning takes place in an iterative manner and is thus independent of change of human's parameters in a single iteration.

### 4.2. Assistance as Needed

**Figure 8:**
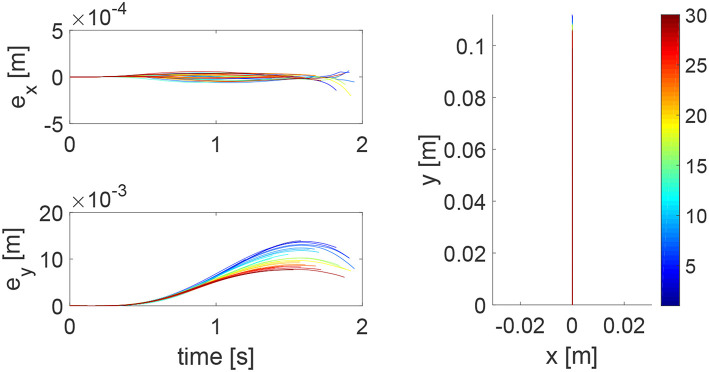
Tracking errors in the *x* direction (top left) and *y* direction (bottom left) and trajectories in the *x* − *y* plane (right column) when the human can partially accomplish the reaching task. The learning process is shown by the color bar, which changes from blue (iteration number *i* = 1) to red (*i* = 30).

**Figure 9:**
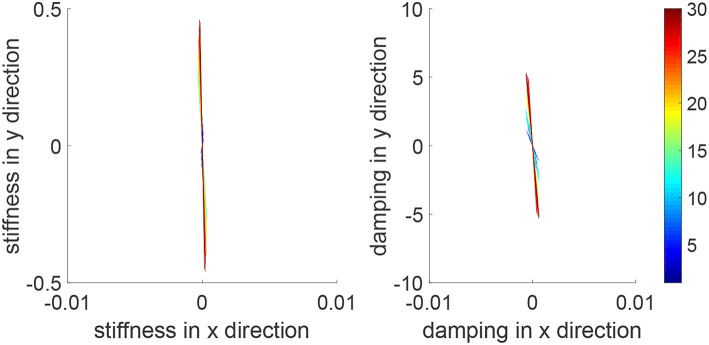
Stiffness ellipse (left) and damping ellipse (right) in all iterations when the human can partially accomplish the reaching task. The learning process is shown by the color bar, which changes from blue (iteration number *i* = 1) to red (*i* = 30).

Assistance-as-needed is an important property in physical training for humans' learning. In this simulation, we show that this property can be realized by the proposed robot controller. In particular, we assume that the human has an ability to accomplish the reaching task partially, instead of destabilizing the system in the previous simulation. Therefore, we set *K*_*h*1_ = 100 × 1_2_ and keep other parameters the same. Simulation results are given in Figures 8, 9. Figure 8 shows that the reaching performance is improved iteratively, with the initial performance much better than that in Figure 3. By comparing Figures 4, 9, we find that the impedance parameters in the latter figure are smaller, suggesting that the robot provides less assistance to the human as the human has a better performance. These results demonstrate that this learning method enables the robot to automatically change its control input that provides assistance-as-needed to the human.

### 4.3. Tracking of a Circular Trajectory

**Figure 10:**
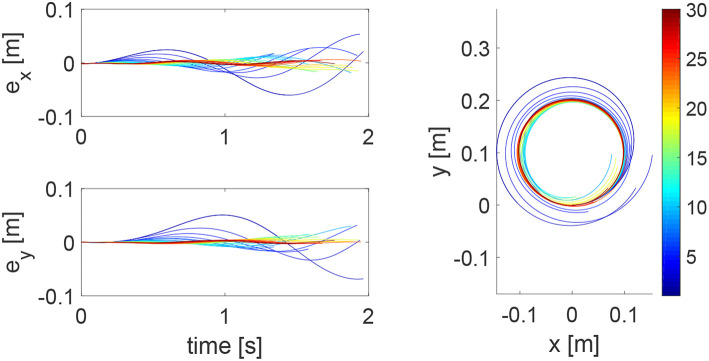
Tracking errors in the *x* direction (top left) and *y* direction (bottom left) and trajectories in the *x* − *y* plane (right column) when tracking a circular trajectory. The learning process is shown by the color bar, which changes from blue (iteration number *i* = 1) to red (*i* = 30).

**Figure 11:**
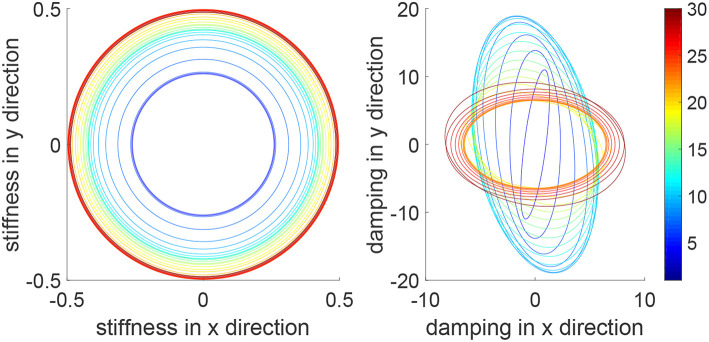
Stiffness ellipse (left) and damping ellipse (right) in all iterations when tracking a circular trajectory. The learning process is shown by the color bar, which changes from blue (iteration number *i* = 1) to red (*i* = 30).

In this simulation, we consider a task where the human and robot need to track a continuous trajectory. Figure 10 shows iteratively improved tracking performance in each direction and tracking of a circular trajectory is achieved after 30 iterations. Figure 11 shows the impedance parameters with changing values in both x and y directions, as the task includes tracking in both directions. Due to similar motions in two directions, i.e., sine and cosine waves, the stiffness ellipse is close to a circle. This is different from that in Figure 4, where there is little motion in the x direction so there is a little change of the impedance parameters in the x direction. These comparative results further demonstrate the assistance-as-needed property of the learning method which not only handles variance of human parameters, but also variance of system settings.

### 4.4. Comparison With Impedance Control

**Figure 12:**
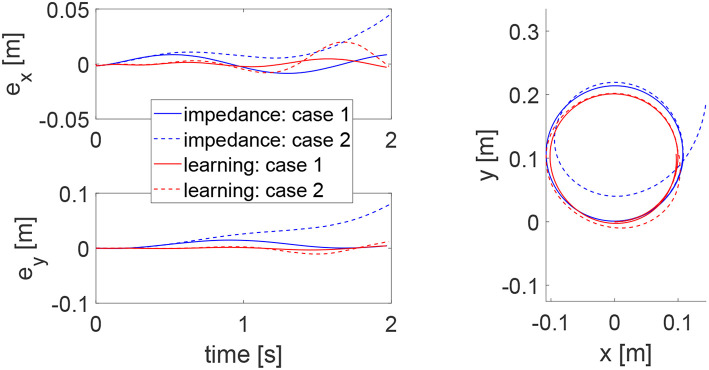
Tracking errors in the *x* direction (top left) and *y* direction (bottom left) and trajectories in the *x* − *y* plane (right column) using impedance control with fixed parameters and the proposed method in Cases 1 and 2. Results in Cases 1 and 2 are respectively represented by solid and dotted lines, while impedance control and the proposed learning method, respectively by blue and red lines.

**Figure 13:**
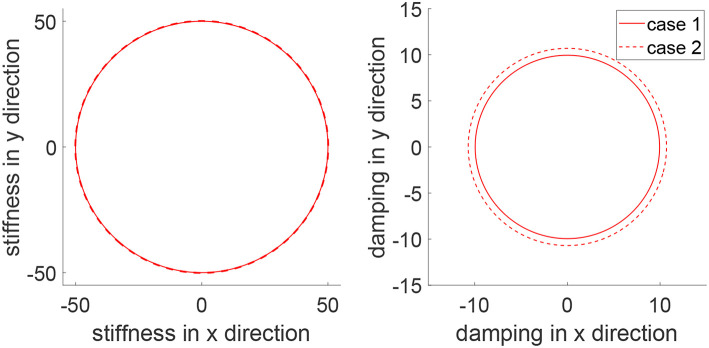
Stiffness ellipse (left) and damping ellipse (right) in Cases 1 (solid line) and 2 (dotted line).

To demonstrate the advantages of the proposed method, we compare it with impedance control with fixed impedance parameters which is a method that is widely adopted in robot-assisted physical training. Two cases are considered where the human's controllers are different, emulating uncertain human behaviors. In Case 1, the human's control parameters are set as *K*_*h*1_ = −50 × 1_2_ and *K*_*h*2_ = −10 × 1_2_, and in Case 2, they are respectively changed to *K*_*h*1_ = −300 × 1_2_ and *K*_*h*2_ = −15 × 1_2_. In both cases, impedance control is implemented as *u*_2_ = −50*e* − 10ẋ and the proposed learning method has the same parameters as mentioned above except initializing the impedance parameters as ${\hat{K}}_{h1}=-50\times 1_{2}$ and ${\hat{K}}_{h2}=-10\times 1_{2}$. The tracking errors in two directions and trajectory under two methods are shown in Figure 12. It is found that impedance control ensures tracking in Case 1 with fine-tuned parameters but it fails to track the circular trajectory when the human's parameters change in Case 2. In comparison, the proposed learning method guarantees small tracking errors and accurate tracking in both cases, as it automatically updates impedance parameters as in Figure 13. These results illustrate that the human uncertainties can be handled by the learning method, which is critically useful in physical training.

## 5. Conclusions

In this paper, we studied impedance learning for robot-assisted physical training. Considering that the human dynamics are difficult to identify but a repetitive process is involved, we employed iterative learning control (ILC) for the development of the learning algorithm. A unique issue of human variance in repeating a motion in physical training was addressed by adopting ILC with varying iteration lengths. Learning convergence was proved in rigor and various human behaviors in physical training were simulated.

Compared to existing methods based on measurement or estimation of the human impedance (Rahman et al., 2002; Erden and Billard, 2015), the proposed method is model-free so does not require a training phase. Compared to those based on reinforcement learning (Kim et al., 2010; Buchli et al., 2011), the proposed one guarantees system stability throughout the interaction and is simple to implement. Compared to the methods of transferring human impedance skills to robots (Ajoudani, 2015; Yang et al., 2018), the proposed one does not require any extra human-robot interface, e.g., EMG sensors or haptic devices. Although with these advantages, it is noted that the proposed method is applicable to only repetitive tasks, which is an assumption that cannot be met in many other applications. In our future works, we are interested in testing this method in a more complicated scenario where a part of the task can be deemed as repetitive, e.g., object loading and offloading. We will also apply the proposed method to real-world physical training and evaluate how it promotes patients' learning.

## Data Availability

The data that support the findings of this study are available from the corresponding authors upon reasonable request.

## Author Contributions

YL, XZ, and XL: control concepts. YL and XZ: algorithm and simulation. YL, JZ, and XL: results analysis. YL, XZ, JZ, and XL: manuscript writing. All authors have read and edited the manuscript, and agree with its content.

### Conflict of Interest Statement

The authors declare that the research was conducted in the absence of any commercial or financial relationships that could be construed as a potential conflict of interest.
